# Sea-Voyages for Invalids

**Published:** 1906-03-17

**Authors:** 


### SEA-VOYAGES FOR INVALIDS.

In the course of a communication on this subject, Dr. Robert Felkin [1] observes that increasing experience renders him less inclined than he used to be to advise sea-voyages promiscuously for invalids, chiefly on account of the altered conditions obtaining on ship-board. "The rush of every-day life, the excessive luxury of ocean liners, the greater number of passengers, the dressing, dancing, the excitement and unrest which obtains on board, are to my mind not so conducive to the rest and regaining of bodily and mental tone as the quieter, lazier, and more simple mode of life on board ship as it was, say, twenty years ago." In particular he considers that phthisical cases should not be sent to sea at random, since the risk of infecting others is too great to be undertaken. He instances the case of two ladies who took, under his advice, a voyage to Australia for the relief of pulmonary tuberculosis in one of them. In this case, although the sufferer gained some temporary relief, the sister and also another cabin companion became infected, and all three died within a year of their return to this country.

In choosing a trip for a patient, when a trip is decided upon, the author insists upon the wisdom of finding out beforehand as much as is possible as to the quality of the ship's doctor, since many ship'sdoctors are young and inexperienced men, chiefly bent upon enjoying their holiday. As to the advisability of recommending a sea-voyage in any given case, weight must be given in the first place to personal idiosyncrasy. Thus it is obviously unwise to send to sea a person who is known to be a bad sailor, whether on the score of sea-sickness or sleeplessness, or constipation while at sea. Further, unless the invalid can travel with a companion, it is often wiser to keep him at home. But supposing none of these disqualifications exist, there are certain physical and mental conditions which render a voyage inadvisable. These are: (1) weakness, a person suffering from great exhaustion is better on land; (2) grave dyspepsia; (3) hepatic enlargement; (4) cardiac dilatation; (5) pyrexia; (6) any tendency to hæmorrhage; (7) epilepsy (8) insanity; (9) pregnancy; (10) eye disease; (11) kidney disease; (12) phthisis, except in the very earliest stages, and then the patient should occupy a deck-cabin *alone*.

The disorders most likely to obtain benefit from a sea-voyage are, in the author's opinion, the following: Firstly, overwork, worry, and mental irritability generally are remarkably benefited by the sea, and that without any great prolongation of the voyage. Thus, for these cases, a trip to the West Indies, or to the Mediterranean, or to the Norwegian, Danish, or Swedish waters in the summer will often prove sufficient—say a trip of a month or six weeks. For certain mild cases the short trip to Madeira or Teneriffe may also be recommended. In graver instances the voyage may be prolonged to the Cape, and include a short stay there, but it is seldom necessary to prescribe so long a trip as that to Australia or New Zealand. Except for those who simply need distraction, tourist-boats are not to be advised, for too much sight-seeing is no good occupation for an invalid. Among other sufferers, those who profit most from voyages, long or short as the case may be, are those affected with bronchitis, emphysema, asthma (except the nervous variety), hay-fever, laryngeal catarrh, rheumatism, gout, scrofula, syphilis, irritable bladder, and nervous diseases such as melancholia, chorea taken early, neuralgia, and hysteria.

The best seasons for voyages to the commoner localities are as follows:—

To the Cape a patient may be sent at any time, though it is best to avoid arrival there in December and January, which are the hottest months. For the Mediterranean the best season is from September to May. For Norway and Sweden, June to September. To India, a patient should be sent so as to spend the months from December to February in that country. The voyage to China or Japan should be undertaken in November. That to the West Indies from October to January. That to Rio, in May or June, but not later. The journey to Australia, New Zealand, or Tasmania should be made *via* the Red Sea, between October and March, but at all other seasons *via* the Cape. In any case it is advised that full instructions should be given as to the clothing required for the climatic conditions likely to be encountered, since public ideas on the subject are very hazy.

The author concludes his paper with a reference to the suggestion that ships should be found for invalids particularly. This suggestion is being put into practice by the Hamburg-American Packet-Steamer Company, which is organising a steamer, to be called the "Fürst Bismarck," as a floating sanatorium. The experiment is to be made under competent medical supervision, and the author suggests that some British company should follow the example. In that case he considers that sea-voyages for invalids would be more successful than they are at present except for a very limited class of cases.

[1] Journ. of Balneology and Climatology, Jan., 1906.

